# Slip considered path planning of a novel angled-spoke based robot in a terrain mixture of granular media and rigid support

**DOI:** 10.1038/s41598-023-49407-4

**Published:** 2023-12-11

**Authors:** Kyungwook Lee, Chaewon Kim, Sijun Ryu, TaeWon Seo

**Affiliations:** School of Mechanical Engineering, Hanyang University, Seoul, 04763 South Korea

**Keywords:** Mechanical engineering, Engineering

## Abstract

Navigation in terrains mixture of rigid support and granular media is associated with slippage. It is important to find the optimal path because slippage implies the possibility of a mobile robot being stuck in the sand. This research introduce concept of effective distance which consider effect of slippage to present a path planning algorithm. Effective distance is a conceptual distance that the mobile robot traverse to reach next node, and it is calculated based on traversable difficulty. Traversable difficulty varies with the slope angle, and the revolution speed of the angled spoke-based wheel (ASW) is estimated using meta-model. The meta-model is achieved empirically. Experiments have done in testbench which can implement slope angle and terrain types. Effectiveness of this algorithm was verified through simulation. For the simulations three cost determination methods, namely, the effective distance with varying revolution speed of the ASW, the effective distance with fixed revolution speed of the ASW, and actual distance were examined in four different scenarios. The effective distance with varying revolution speed of the ASW has maximum $135.94\%$ longer total actual distance. However, it results minimum $44.72\%$ shorter total effective distance.

## Introduction

Mars rover Opportunity was trapped in loose granular media terrain, and it took five weeks to free it and return to the mission. NASA’s Mars Exploration Rover Spirit entrapped in granular media, leading to mission failure^1,2^. These accidents indicates that operation of a mobile robot in an environment containing granular media is challenging. A granular medium appears in landslide disasters and exists in the regoliths of Mars or the Moon. The topographical characteristic of these environments have a unique characteristic that they can exhibit solid-like and fluid-like behavior because of granular media^3^. Moreover, rigid support can be interpreted as perfectly solid-like behaving granular media under any conditions when mobile robots drive. Therefore, to explore these environments, mobile robots should be traversable on both granular media and rigid support terrains.

Successful attainment of the target for the mobile robot in granular media requires not only locomotability but also ability to search for an adequate path. Unability of searching adequate path leads to failure of the mission. Path planning is process of computing an optimal path from the current position to the desired goal. Granular media are a challenging terrain for mobile robots to locomote^4^ because it causes slippage between wheels and terrain. Mobile robots can experiences slippage even cab be entrapment in such terrains. Entrapment is an anomalous situation in which a robot experiences nearly $100\%$ slippage^5^.If entrapment occurs, the robot cannot advance in any direction but is trapped. The presence of slippage affects the timeline of missions or implies the possibility of mission failure. Therefore, it is crucial to diagnose slippage to find the optimal path in a terrain containing granular media.

Future exploration missions in terrains containing granular media will demand reliable slippage estimation and compensation strategies for safer and more efficient navigation^5^ beyond distinguishing traversability. Studies have been conducted to find the optimal global path of mobile robots in granular media. Defining cost function based on interaction between wheel and terrain was proposed. Hua et al. focused on deformation of soil due to force applied to wheels^6^. Methods of classifying terrain types to differ cost applied while the robot traverse each terrains. Ono et al. used terrain classification to identify risk of traveling each terrains including effect of slope^7^, and Inotsume et al. include effect of surface condition such as compaction level and accumulated depth^8^. Learning based method were also studied. Yu et al. introduced learning based path planner using Markov decision process with physical engine^9^. However, those researches does not consider slippage caused by angular velocity of wheels.

The main contribution of the research is presenting algorithm to find the optimal path which causes the least slippage for a mobile robot. The algorithm was generated based on the mobile robot with angled spoke-based wheels (ASWs) which is validated for traversing in granular media and rigid support terrain^10^. Angular velocity of ASW is couple with slippage, and it determines traversing time to the target. While lower angular velocity of ASWs causes less slippage of the mobile robot, it increases traveling time to the target. Therefore, it is crucial to find adequate angular velocity to minimize slippage. The optimal angular velocity of ASW varies with terrain types and slope angle. Though studies have conducted to model behavior of granular media with varies methodologies, mathematical model to describe behavior of granular media affected by shape and angular velocity of ASWs varies with slope angle have not established^11^. Thus, slip ratio of ASW in rigid support and granular media is formulated empirically. Then simulated optimal path is compared with other paths which does not consider slip ratio or effect of angular velocity of ASW.

## Robot design and characteristic

### Robot design

**Figure 1:**
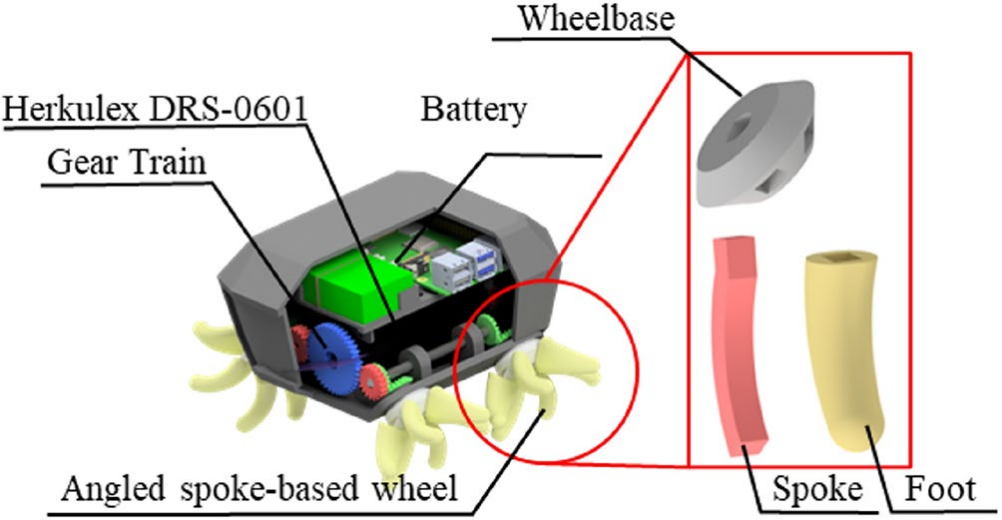
Configuration of DODO and angled spoke based wheels.

DODO, a mobile robot with four angled spoke-based wheels (ASWs), is capable of obstacle overcoming and locomoting multiple terrains including granular media and rigid support^10^. Configuration of DODO and ASWs are shown in Fig. 1. It advantage with compacted size and agile locomotion from simple mechanism. Agility of locomotion in granular media was assured by optimizing configuration of foot^12^, and traversability in both granular media and rogod support have been verified^13^.

The design of the robot with ASWs is illustrated in Fig. 1. The robot consists of a body and four ASWs. The dimensions were 180 mm $\times$ 120 mm $\times$ 120 mm, and the body weight was 1030 g. Acrylonitrile styrene acrylate (ASA) material was used to construct the robot body. Two servo motors (Herkulex DRS-0601) were used to move and steer the robot. Two ASWs in each side is connected to rods through 1:1 bevel gears to generate $45\,^{\circ }$ tilted degree to horizontal plane. Rods are connected to servo motors using 1:1 spur gears. Power can be transmitted from servo motors to ASWs in each side. Controlling speed and direction of each motors allow the robot to steer and moving back and forth. The internal gearbox of the motors was switched to increase the motor velocity by approximately 6.5 times. Motor velocity was measured in units of revolution per minute (rpm). A Raspberry Pi 4 was used to control the speed and steering of each motor by serial communication.

The configuration of the ASW is based on the optimization results from previous studies^12,13^. Spokes with ASA material were covered with a 3 mm thick silicone (VytaFlex 60) material cover. Soft material (silicone) was used to increase the friction between the spoke and rigid support and reduce the impact when it contacts terrains. Spokes consisted with ASA material were inserted center of Silicone foot. Without rigid material, soft material got bent and remains difficult for a spoke to maintain rigidity while a mobile robot is moving.

### Characteristic of ASWs

Avoiding slippage is one of the most important factors for a successful drive for an ASW in a multi-terrain. When the robot locomotes on rigid support, the rotating speed of the ASWs generates impact on the terrain. This impact creates a reaction force when the robot is in contact with rigid support. The reaction force reduces the normal force and leads to a reduction in friction. Because an ASW moves through a series of discontinuous spokes contacts, reduced friction causes slippage.

Slippage occurring while driving in granular media is even more crucial. Wheel speed is a representative factor that alters the characteristics of a granular medium^14^. When the granular medium is fluidized, it causes slippage. Slippage slows down the speed of the robot. Moreover, the robot could get stuck in the sand, and this study identifies this as sinkage. When sinkage occurs, it leads to mission failure.

**Figure 2:**
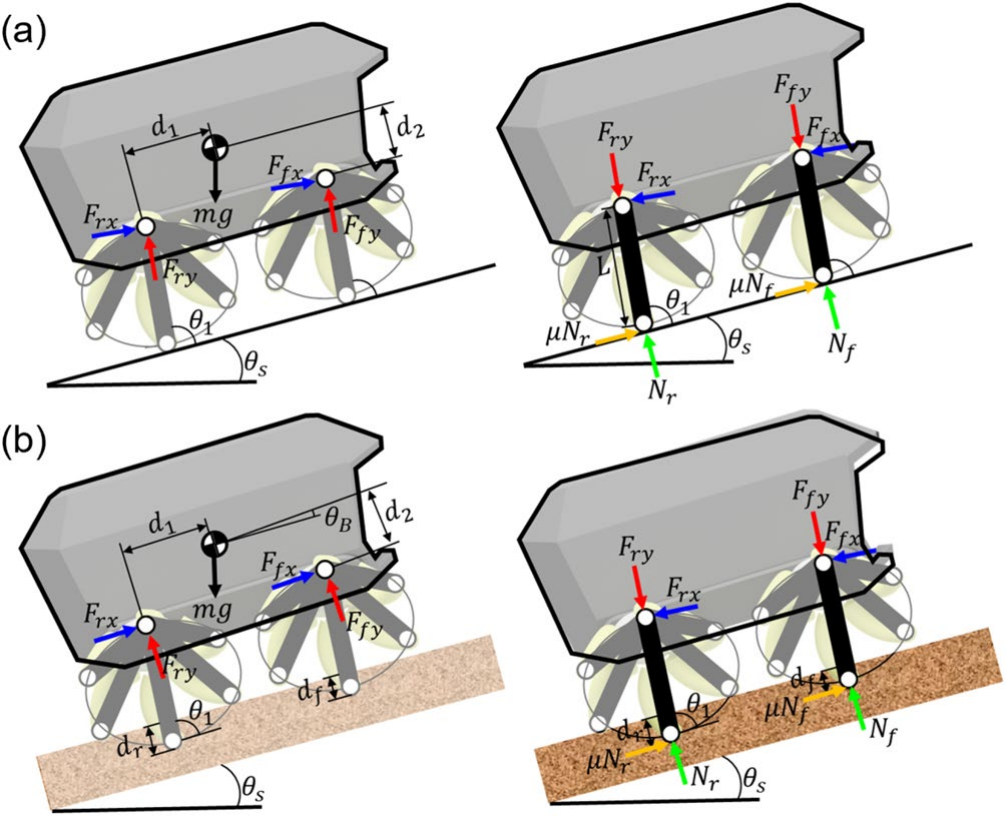
Static analysis of DODO on (**a**) Rigid support and in (**b**) Granular media when traversing sloped angle.

The static behaviors of ASWs on rigid support and granular media are shown in Fig. 2. The front and rear ASWs are synchronized, and the distances between the center of mass and the axis of each front and rear ASW are identical. The normal forces of the front and rear spokes,$N_f$ and $N_r$, on rigid support are represented as follows:

1 $$\begin{aligned} \begin{aligned} N_r&=\frac{(d_1+d_2\mu _g)}{2d_1}\times \frac{1}{2}\ mg\times cos\theta _s \\ N_f&=\frac{(d_1-d_2\mu _g)}{2d_1}\times \frac{1}{2}\ mg\times cos\theta _s. \end{aligned} \end{aligned}$$

$F_{fx}$ and $F_{rx}$ are force applied to *x* axis of front and rear wheel base, and $F_{fy}$ and $F_{ry}$ are *y* axis force applied to front and rear wheel base because of $N_f$ and $N_r$. $d_1$ and $d_2$ are the *x* and *y* distances between the axis of rotation of the ASW and center of mass, respectively. *m* denotes mass of the robot, and *g* is gravitational acceleration. $\mu _g$ is the dry friction coefficient on rigid support. As the slope $\theta _s$ increased, the normal force decreased. When the robot climbed up the slope, the normal force of the front spoke was smaller than that of the rear spoke. Therefore, smaller normal force of front spoke pivots at the rear spoke and is flipped backwards.

Unlike its behavior on rigid support, the ASW penetrated the granular media. The penetration balances the body weight and momentum applied to each spoke^15^. The generated body angle was not parallel to the slope angle. The force applied to the spoke determined the penetration depth. The normal force applied to each spoke was calculated as follows:

2 $$\begin{aligned} \theta _B= & {} \sin ^{-1}{\left( \frac{d_r-d_f}{2d_1}\right) } \end{aligned}$$

3 $$\begin{aligned} \alpha= & {} \left( \frac{\mu _s sin\theta _B-cos\theta _B}{\sin ^2{\theta _B}-\cos ^2{\theta _B}}\right) \nonumber \\ \beta= & {} \left( \frac{sin\theta _B-\mu _s cos\theta _B}{\sin ^2{\theta _B}-\cos ^2{\theta _B}}\right) \end{aligned}$$

4 $$\begin{aligned} N_f= & {} \left( \frac{d_1\alpha -d_2\beta }{2d_1\alpha ^2}\right) \times \frac{1}{2}mg\theta _s \nonumber \\ N_r= & {} \left( \frac{d_1\alpha +d_2\beta }{2d_1\alpha ^2}\right) \times \frac{1}{2}mg\theta _s. \end{aligned}$$

$\theta _B$ denotes the body angle, $d_r$ and $d_f$ are the penetration depths of the front and rear spokes, respectively. $\mu _s$ is the dry friction coefficient in granular media. $\alpha$ and $\beta$ are unknown terms to tidy the equation.

## Path planning algorithm

### $\hbox {A}^{*}$ algorithm

Path planning is a method of moving robots from one point to another. This research was conducted using $\hbox {A}^{*}$ algorithm which is one of the well known graph-based path planning algorithms. This is because graph-based algorithms have advantage of small number of required sample and memory size over sampling based algorithms such as $\hbox {rrt}^{*}$^8,16^. Indeed, graph-based path planning can find the optimal path. In order to compare influence of cost function that affects generating path to the goal under same condition, all paths should be the optimal path. Therefore, $\hbox {A}^{*}$ algorithm is used in this research.

$\hbox {A}^{*}$ algorithm examine possible paths by combining a heuristic search and node-to-node cost search.

5 $$\begin{aligned} \begin{aligned} f(\nu )=h(\nu )+g(\nu ). \end{aligned} \end{aligned}$$

The nodes that the robot searches are called search nodes. Heuristic distance $h(\nu )$ is calculated as simple norm between one of the searching nodes to the goal node. $g(\nu )$ is a cost function which is a physically meaningful measure between current node to searching node. Optimal path is found in a way of minimizing $f(\nu )$. $g(\nu )$ from the mother node is inherited and accumulates.

The search, current, and goal nodes are expressed as $n_s$, $n_c$, $n_g$ respectively. $n_s$ denotes the number of nodes that the robot can advance from $n_c$. This algorithm calculates $f(\nu )$ for every $n_s$ based on $n_c$. Once $f(\nu )$ is calculated, it is added to the list, which is called an open list containing an information of its mother node. The mother node is $n_c$ of $n_s$. The next node that the robot will move to is the node with the lowest $f(\nu )$ in the open list, which is added to the closed list and deleted from open lists. If the newly calculated $f(\nu )$ of a certain node is smaller than that in the open list, $f(\nu )$ and mother node are swapped to the new value. This search ends when it reaches the goal node $n_g$.

#### Definition of the cost function

The optimal path is obtained by searching for and selecting nodes to minimize optimality, which is quantified as a physically meaningful measure. This optimality is known as the cost function. This research defines the cost function as an effective distance. Effective distance is a conceptual distance that DODO should travel to reach next nodes. If direction of DODO and slip are in same direction, effective distance gets shorter otherwise, it gets longer.

Slippage is the most influential feature to estimate number of revolution. Slippage hinders advancement of the robot under same number of rotation. Therefore, the robot should compensate distance which the robot could not advance due to slippage. Slippage increases number of revolution of ASWs. However, slippage does not always hinder the driving ability of an ASW. When slippage is applied in the same direction as the robot advances, it reduces the revolution number of the ASWs. Slippage is usually defined by the slip ratio *S*^17^, calculated as follows:

6 $$\begin{aligned} \begin{aligned} S=1-\frac{v_{actual}}{v_{ideal}}. \end{aligned} \end{aligned}$$

$v_{actual}$ is an actual driving speed of the robot and it is calculated by measuring traveling speed of the robot when it travel unit distance. This will be addressed detail in Section C. $v_{ideal}$ is an ideal driving speed of the robot. Ideal distance that ASW can travel per revolution under assumption of non-slip condition, and $v_{ideal}$ can be calculated by multiplying angular velocity to ideal distance.

The robot only needs revolution number *n* of ASWs to reach the target under non-slip condition. However, under slip condition required revolution number got changed to $n_{slippage}$. Distance that the robot can advance with $n_{slippage}$ under non-slip condition is defined effective distance. The shortest effective distance stands for the minimum number of revolution of ASWs. The effective distance ${\hat{d}}$ is defined as follows:

7 $$\begin{aligned} \begin{aligned} {\hat{d}} = \frac{1}{1-S}\times d \end{aligned} \end{aligned}$$

*d* is the actual distance between two nodes.

The effective distance can be interpreted as the distance multiplied by traversable difficulty $\frac{1}{1-S}$. Therefore, the cost function can be calculated if the traversable difficulty is estimated. The traversable difficulty models have achieved empirically because deriving the dynamic behavior of an ASW in granular media mathematically is challenging.

### Experimental setup

**Figure 3:**
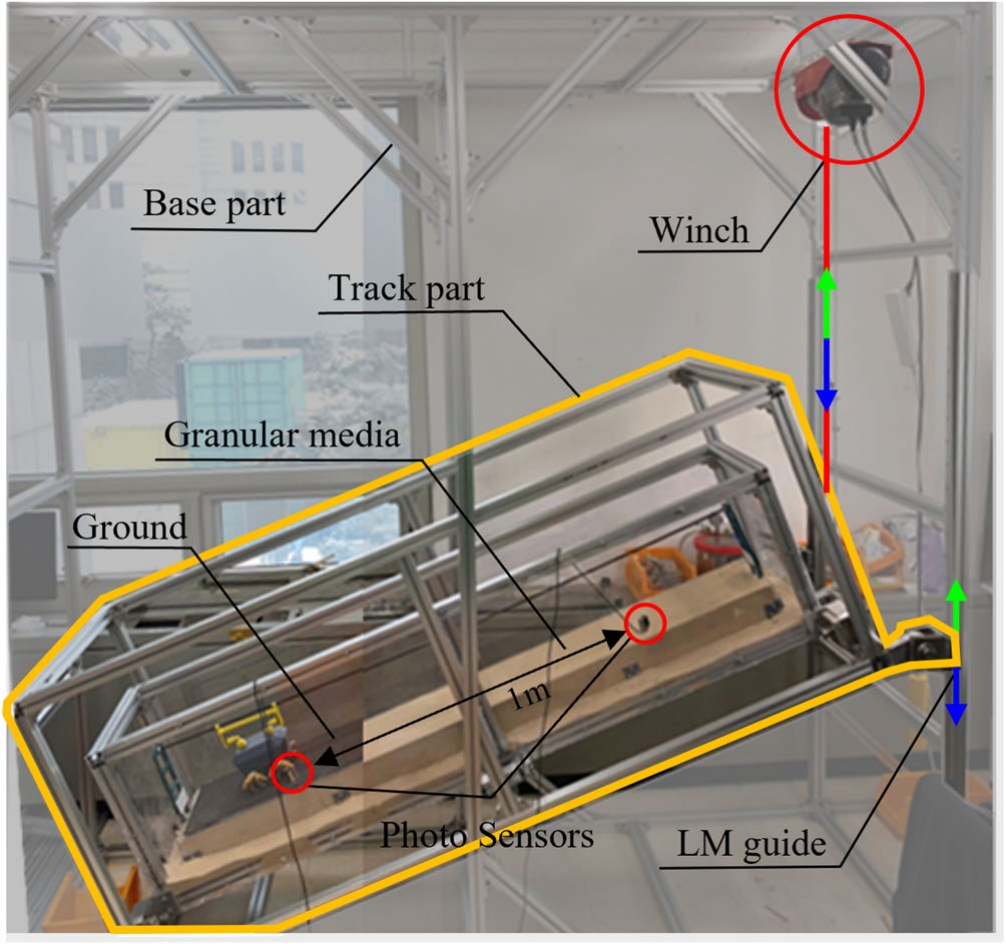
Testbench setup for empirical modeling of the traversable difficulty model.

The slippage is influenced by the slope angle that the robot faces in a 3D environment and the revolution speed of the ASW. Since the difficulty of mathematical model which describes behavior of DODO in granular media, effect of slippage should be achieved empirically. Therefore, A test bench which can implement slope angle of granular media and rigid support was constructed, as shown in Fig. 3. The test bench consisted of two parts: a track and base. The base implements the slope of the terrain using linear guides and a winch (UW300-12). The winch drags the side of the outer box upward to generate a slope. The track part implements the terrain environment. rigid support was created by attaching a urethane surface to an acrylic board, and granular media was created using river sand. Carbon pipes with 6 mm radius were placed at the center of the track to maintain the straightness of the robot’s driving direction. The robot was connected to a carbon pipe with two connectors attached to the front and rear of the body. Two small linear guides were placed on the connectors to allow for pitch angle rotation. Two photo sensors were attached at a 1 m distance. These sensors measured the average speed of the robot at each experimental point and the average velocity of the robot.

### Meta models

**Figure 4:**
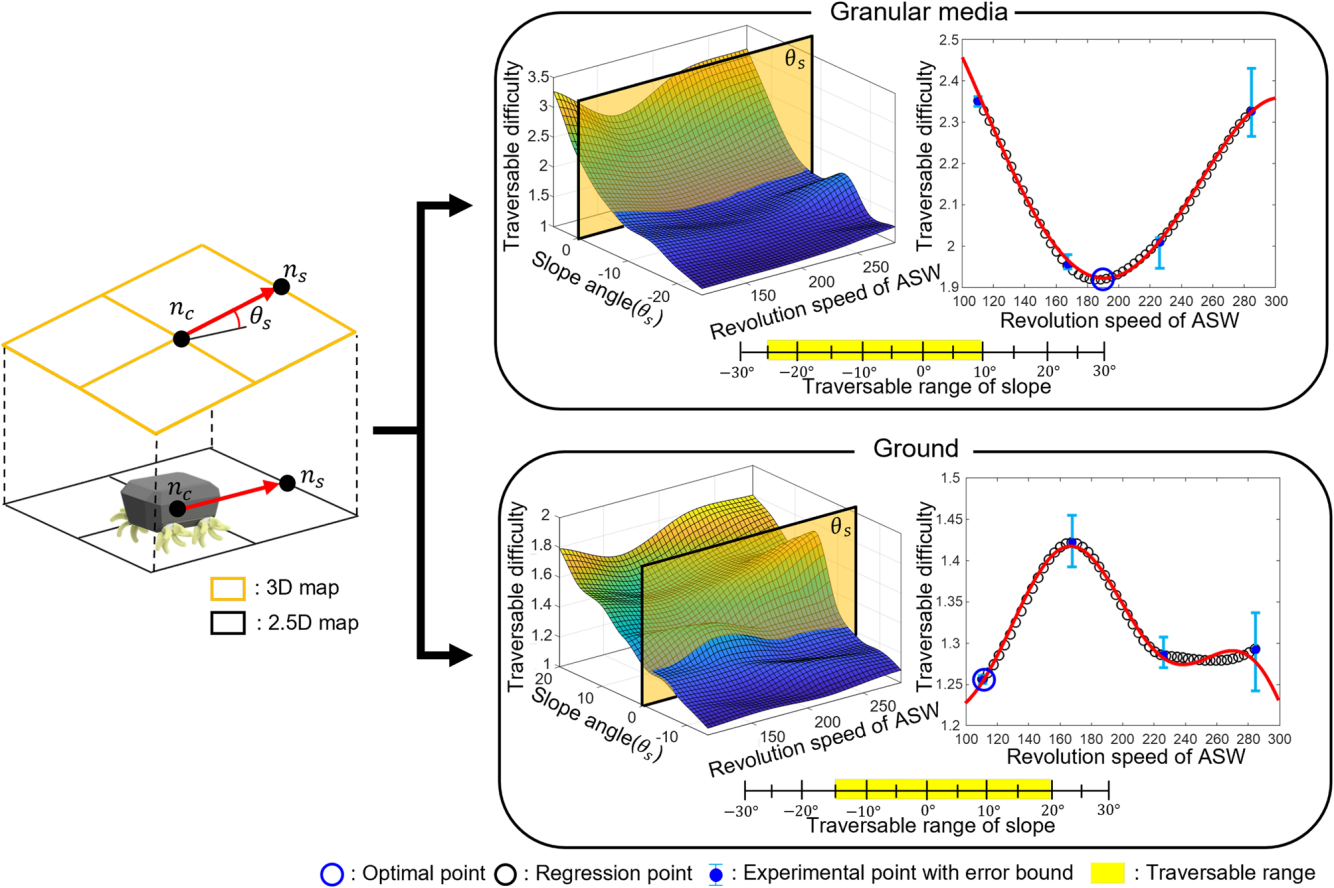
Interpolated (left) and regressed (right) meta-models in granular media (top) and rigid support (bottom).

In order to calculate $g(\nu )$ two meta-models are required, as shown in Fig. 4. The first meta-model was achieved by interpolating the experimental points. A total of 32 experimental points were selected for each terrain and traversability data are shown in Tables 1 and 2. Interpolation was performed with a 3rd order spline method for both granular media and rigid support. The benefits of achieving the slip ratio vary with the motor speed at a certain degree of slope for this interpolated meta-model.

The second meta-model was a regressed model in which the traversable difficulty varied with motor speed. Once slope angle $\theta _s$ was determined, 50 equally distributed points of revolution speed of ASW between 110.41 (rpm) to 284.26 (rpm) and traversable difficulty values at the speeds were extracted. Based on the extracted point regression, a summation of the sinusoidal function was conducted. The minimum value of the traversable difficulty and revolution speed of ASW were estimated. These values were applied to calculate the effective distance for the robot to move to the next node.

### Searching algorithm

Every iteration algorithm searches traversable nodes and calculates their cost. The algorithm searches eight adjacent nodes and examines traversability. The robot can only locomote across granular media terrain with a slope range of $-\,25^{\circ }$ to $10^{\circ }$ and rigid support terrain with $-\,15^{\circ }$ to $20^{\circ }$. Nodes with a slope out of the range are considered non-traversable nodes and are excluded from the search. Once traversable nodes are selected, the slope angles of each node are applied to the meta-models to estimate the minimum traversable difficulty called the optimal point as shown in Fig. 6. The effective distance was calculated by multiplying the traversable difficulty by the actual distance of $n_c$ by $n_s$. The actual distance was calculated using the norm of the two nodes.

## Simulation

### Map design

Path planning was conducted in a 2.5D grid-based map. Grid is drawn by equally distributing *x* and *y* coordinates. Each intersections of grid are nodes. Owing to the computational complexity of 3D map path planning, it has the disadvantage of slow computational speed compared to 2D grid maps. The 2.5D grid is a type of 2D grid map that contains elevation information for every node. Each node on 2D map contains height information, as well as *x* and *y* coordinates. A 3D map can also be approximated by interpolating each node. The slope can also be calculated from the elevation information of the two nodes. Therefore, a 2.5D grid based map is suitable for path planning.

**Table 1:** Traversable difficulty in granular media.

Slope angle [$^{\circ }$]	Angular velocity[rpm]			
	400	600	800	1000
− 25	1.11	1.12	1.15	1.26
− 20	1.26	1.20	1.23	1.25
− 15	1.49	1.39	1.40	1.76
− 10	1.55	1.38	1.40	1.51
− 5	1.83	1.54	1.58	1.68
0	2.35	1.95	2.01	2.33
5	3.27	2.58	3.04	3.10
10	4.17	6.13	14.22	19.48

**Table 2:** Traversable difficulty on rigid support.

Slope angle [$^{\circ }$]	Angular velocity[rpm]			
	400	600	800	1000
− 15	1.05	1.06	1.05	1.08
− 10	1.13	1.09	1.18	1.15
− 5	1.22	1.22	1.16	1.16
0	1.26	1.42	1.29	1.29
5	1.40	1.34	1.55	1.75
10	1.54	1.46	1.56	1.57
15	1.59	1.48	1.59	1.70
20	1.79	1.64	1.80	1.80

**Figure 5:**
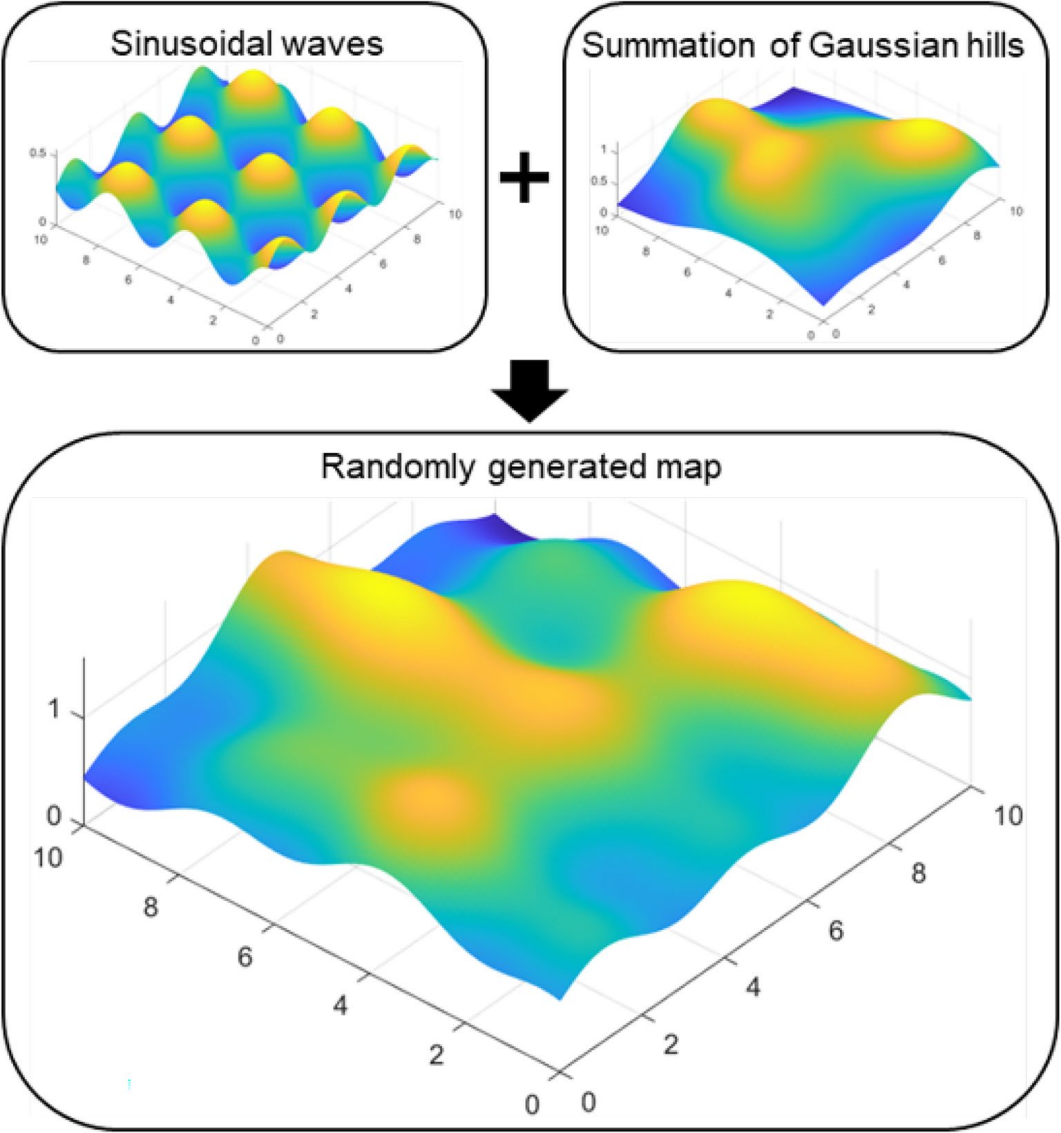
Randomly generated map for simulation.

Maps were formulated, as shown in Fig. 5. They constitute a combination of base and hill patterns. The base pattern was formed using three-dimensional (3D) sine waves. This is formulated as follows:

8 $$\begin{aligned} \begin{aligned} z_{sinusoidal}=sin(x)+cos(y). \end{aligned} \end{aligned}$$

25 randomly generated Gaussian hills were used to form the hill pattern. Gaussian hill has been used as a initial condition of sand dune to estimate movement of it^18^. Moreover, Gaussian hill allows generating continuous map to avoid unnatural geological features. The Gaussian hills are formulated as follows:

9 $$\begin{aligned} z_{gaussian}= & {} \frac{A}{2\pi \sqrt{\sigma _x\sigma _y}}\times \exp ^{-\frac{(x-x_o)^2}{2\sigma _x}-\frac{(y-y_o)^2}{2\sigma _y}} \end{aligned}$$

10 $$\begin{aligned} \sigma _x,\sigma _y >= & {} 0.34\times A \end{aligned}$$

**Figure 6:**
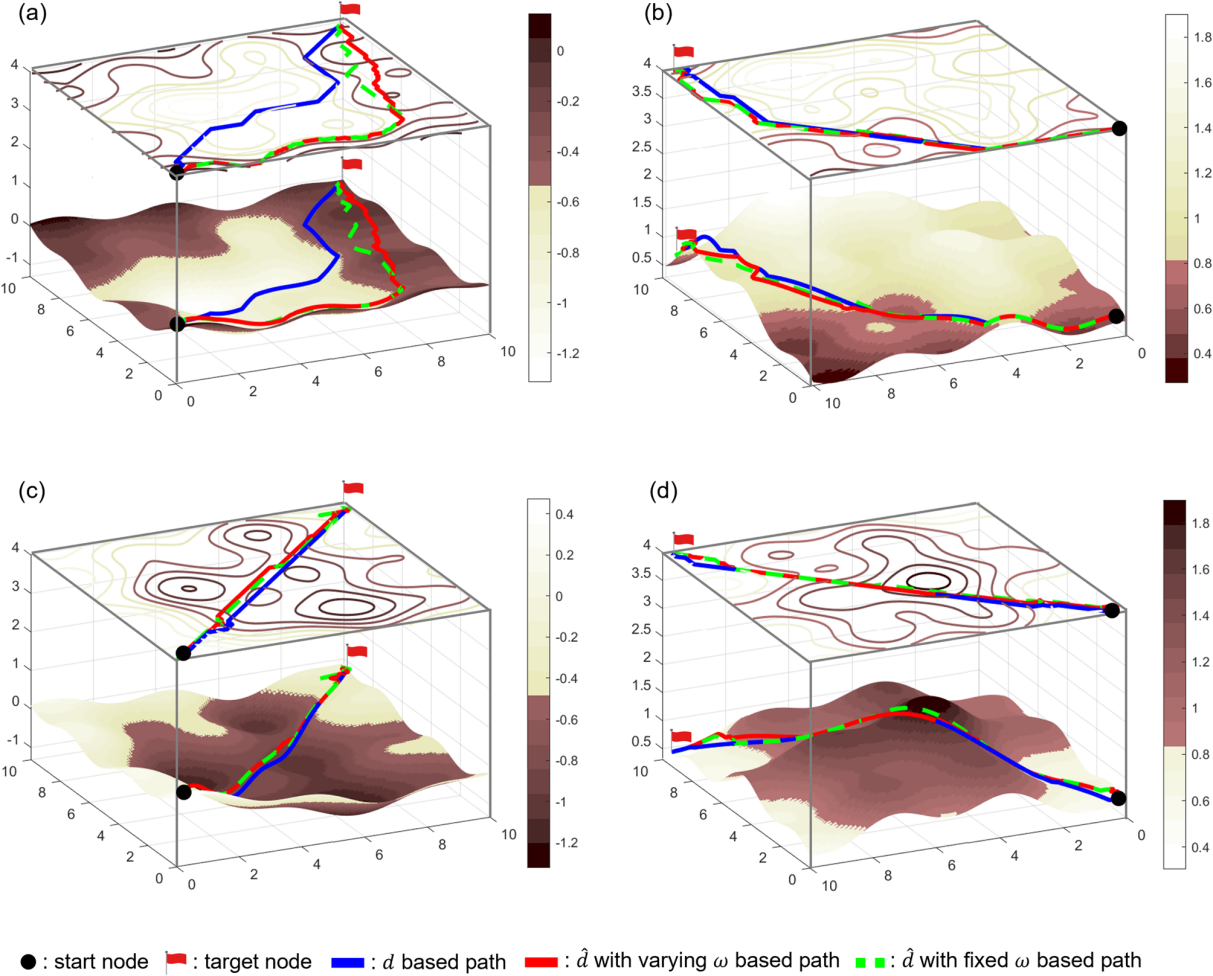
Simulation maps and results. (**a**) Granular media centered basin, (**b**) Granular media centered hill, (**c**) Rigid support centered basin, (**d**) Rigid support centered hill.

*z* denotes the elevation, and *x* and *y* are the coordinates of the node. The mean and variance of *x* and *y* coordinate, and height are represented as $x_0$, $y_0$, $\sigma _x$, $\sigma _y$, and *A*, respectively. The grain size of the river sand used for the experiment was between 0.06 mm and 2 mm^19^. River sand sized particles cannot form hills whose slope angle is steeper than $65^{\circ }$^20^. Therefore, the constraint shown in Eq. (10) is applied. An example of this map is shown in Fig. 6. It is meaningful to achieve statistical results because it can represent the diverse appearances of terrains. The map was a $10\times 10\,m^2$ rectangular area. The size of each cell was $0.1\times 0.1\,m^2$.

### Simulation maps

The simulation maps are shown in Fig. 6. Four types of terrains were simulated. The area marked with a light brown color contour is the granular media, and the rigid support is marked with a darker brown. The upper line of the figure shows the traverse path of the robot. It starts from the rigid support terrain, passes through a hill or valley of granular media, and reaches the goal. The bottom line shows the path from the granular media through the rigid support and again reaching the rigid support.

Three paths with cost function of *d*, ${\hat{d}}$ with varying $\omega$, and ${\hat{d}}$ with fixed $\omega$ were tested. The heuristic values of all three algorithms were identical because of the simple distance between the search and goal nodes. The cost of each algorithm is the effective distance with varying revolution speed of ASW, effective distance with fixed revolution speed, and actual distance. The revolution speed was fixed at 284.26 (rpm) for the search algorithm. The results of effective distance with varying revolution speed of ASW, effective distance with fixed revolution speed of ASW, and actual distance are presented as red, dotted green, and blue lines, respectively.

### Result of simulation

The objective of this research is finding the optimal path which requires the least total revolution number of ASW.

The least total revolution number of ASW can be replaced as the least total effective distance. Simulation results are shown in Fig. 6 and Table 3. The effective distance with varying revolution speed of the ASW has maximum $135.94\%$ longer total actual distance. However, it results minimum $44.72\%$ shorter total effective distance. Simulation results shows that algorithm with the effective distance with varying revolution speed of the ASW has the least total revolution number of ASW. Detailed discussion about effects of traversable difficulty and revolution speed of ASW will be followed.

#### Effect of the traversable difficulty

Effective distance-based paths show tendency to choose rigid support nodes rather than granular media nodes, as shown in Fig. 6a. This is because the traversable difficulty of the granular media is higher than that of rigid support. In addition, effective distance-based paths tend to avoid steep uphill slopes while preferring steep downhill slopes. Although this tendency was observed for both rigid support and granular media, it was more significant in case of granular media. This is because the traversable difficulty drops rapidly as the slope angle becomes smaller in granular media as compared to on the rigid support. The traversable difficulty while ascending granular media reached 19.48.

The descending path is relatively similar to the ascending path. This is because as the robot moves downhill, the traversable difficulty approaches 1. Because a simple distance-based path is the path for which the traversable difficulty is 1, as the traversable difficulty reaches 1, optimal path determined by effective distance gets much similar to path determined by actual distance. However, because the slip ratio was still larger than 1, the paths still showed differences.

#### Effect of revolution speed of ASW

The total effective traveling distance for each simulation map is presented in Table 3. The slope angle of each node from the path was used to calculate the total traveling effective distance of the algorithm. Information of traversable difficulty was estimated from the slope. The effective distance was then calculated based on Eq. (7).

As shown in Fig. 6a and Table 3 total actual distance of *d* based path has shorter distance compared to ${\hat{d}}$ based path. However, the results reveal that varying the revolution speed of the ASW results in a shorter effective distance. In addition, effective distance-based methods show shorter total effective distances than the actual distance-based methods. However, except in the case where the robot travels in granular media downhill, all the other cases show that the actual distance-based path with varying revolution speeds has a shorter total effective distance than the path generated based on the effective distance with a fixed revolution speed. Therefore, controlling the revolution speed of ASW affects slippage and effectively reduces the total effective distance required to reach the goal.

**Table 3:** Total effective distance and actual distance.

Simulation map	Effective distance				Actual distance	
	${\hat{d}}$ based algorithm		*d* based algorithm		${\hat{d}}$ based algorithm	*d* based algorithm
	Varying $\omega$	Fixed $\omega$	Varying $\omega$	Fixed $\omega$		
(a)	22.40 m	24.88 m	29.26 m	50.09m	16.27 m	14.15 m
(b)	29.30 m	46.71 m	35.06 m	63.48 m	20.20 m	14.86 m
(c)	21.34 m	24.84 m	22.44 m	29.85 m	18.78 m	14.41 m
(d)	22.52 m	32.81 m	25.18 m	40.76 m	19.58 m	15.25 m

### Supplementary Information

Supplementary Legends.

Supplementary Video 1.

## Data Availability

All data generated or analysed during this study are included in this published article and its Supplementary Information files.
