# Supplementary material for: Slip considered path planning of a novel angled-spoke based robot in a terrain mixture of granular media and rigid support
Source: Sci Rep. 2023 Dec 11;13:21902. doi: 10.1038/s41598-023-49407-4 (PMC10713549; doi:10.1038/s41598-023-49407-4)
Supplement: Supplementary file 1 — Supplementary Legends. [file 41598_2023_49407_MOESM1_ESM.pdf]

Video 1: Multimedia of Slip considered path planning of a novel angled-spoke based robot
